# Retraction Note: microRNA-195 attenuates neuronal apoptosis in rats with ischemic stroke through inhibiting KLF5-mediated activation of the JNK signaling pathway

**DOI:** 10.1186/s10020-023-00650-5

**Published:** 2023-04-13

**Authors:** Lisha Chang, Wan Zhang, Songxin Shi, Yanbo Peng, Dali Wang, Li Zhang, Jiang Zhang

**Affiliations:** 1 Department of Neurology, North China University of Science and Technology Affiliated Hospital, No. 73, Jianshe South Road, Tangshan, 063000 Hebei Province People’s Republic of China; 2 Quality Control Office, North China University of Science and Technology Affiliated Hospital, Tangshan, 063000 People’s Republic of China

**Retraction Note: Molecular Medicine (2020) 26:31**

10.1186/s10020-020-00150-w

The Editor-in-Chief has retracted this article at the corresponding author’s request. After publication, the authors became aware that the presented results could not be reproduced. Further checks by the publisher raised concerns regarding the animal models used in the study (miR-195-knockout and KLF5-knockout male Sprague-Dawley rats), as no information about this model is available from the stated supplier.

All authors agree to this retraction.

